# Thermal Strain During Open-Water Swimming Competition in Warm Water Environments

**DOI:** 10.3389/fphys.2021.785399

**Published:** 2021-12-23

**Authors:** Samuel Chalmers, Gregory Shaw, Iñigo Mujika, Ollie Jay

**Affiliations:** ^1^Alliance for Research in Exercise, Nutrition, and Activity (ARENA), Allied Health and Human Performance, University of South Australia, Adelaide, SA, Australia; ^2^Thermal Ergonomics Laboratory, Faculty of Medicine and Health, The University of Sydney, Sydney, NSW, Australia; ^3^High Performance Unit, Swimming Australia, Brisbane, QLD, Australia; ^4^Department of Physiology, Faculty of Medicine and Nursing, University of the Basque Country, Leioa, Spain; ^5^Exercise Science Laboratory, School of Kinesiology, Faculty of Medicine, Universidad Finis Terrae, Santiago, Chile

**Keywords:** swimming, water temperature, heat, thermal strain, policy

## Abstract

Open-water swim racing in warm water is associated with significant physiological strain. However, existing international policy that governs safe participation during competition relies only on a fixed water temperature threshold for event cancellation and has an unclear biophysical rationale. The current policy does not factor other environmental factors or race distance, nor provide a stratification of risk (low, moderate, high, or extreme) prior to the threshold for cancellation. Therefore, the primary aim of this *Perspectives* article is to highlight considerations for the development of modernized warm-water competition policies. We highlight current accounts (or lack thereof) of thermal strain, cooling interventions, and performance in warm-water swimming and opportunities for advancement of knowledge. Further work is needed that systematically evaluate real-world thermal strain and performance during warm water competition (alongside reports of environmental conditions), novel preparatory strategies, and in-race cooling strategies. This could ultimately form a basis for future development of modernized policies for athlete cohorts that stratifies risk and mitigation strategies according to important environmental factors and race-specific factors (distance).

## Introduction

Open-water swimming was first introduced as a medal event (25 km) at the Fédération Internationale de Natation (FINA) World Championships in 1991 (Shaw et al., 2014). In 2008, the marathon event (10 km) was introduced at the Olympic Games. Since its first appearance in 1991, open-water events have become a major discipline in FINA aquatic sports, including a separate 7-day world championships competition schedule including 5 km, 10 km, 25 km, and 5 km mixed relay events (FINA, 2019). Additionally, yearly marathon and ultramarathon race series are held at numerous locations around the world over a 10-month period. This has led to open-water racing competition becoming extremely diverse from a geographical and environmental perspective.

Currently, FINA has a defined scope of environmental conditions within which an open-water event can be undertaken. Regulations are associated with minimal and maximal water temperatures (T_w_) and quality and include recommendations of swimwear use for T_w_ below 20°C (Fédération Internationale de Natation (FINA), 2020). However, little coordinated attention has been given to interventions and policies in warm T_w_ (≥29°C; Bradford et al., 2013) to underpin the advised maximum temperature threshold. Prior to 2011, there was not a well-enforced maximal T_w_ that defined extreme risk of exertional heat illness. However, a cut-off was developed as a consequence of the tragic heat-related death of open-water swimmer Fran Crippen in 2010, which was attributed to competing in T_w_ above 31°C (Macaluso et al., 2013). A suggested T_w_ threshold for cancellation of 31°C was proposed in early 2011, but even after this recommendation, FINA completed events in environments with documented T_w_ around or above 31°C (Munatones, 2021). Finally, FINA updated T_w_ limits in 2013 to mandate the cancellation threshold of 31°C. However, the biophysical rationale justifying this threshold remains unclear.

Concerningly, numerous open-water races have been reported to be completed in T_w_ above 31°C as non-sanctioned races, or races where the rules have been ignored. For example, the World University Games in Taiwan (Keith, 2017) and the World Beach Games in Doha (Lord, 2019) completed open-water races in T_w_ that was reported to be above the 31°C threshold. Recently, during the Tokyo 2020 men and women’s Olympics open-water marathon swimming competition, the start time was rescheduled for 2 h earlier to ensure T_w_ was below 31°C. The discussion around T_w_ and swimmer health is of significant interest with the upcoming 2023 FINA world open-water championships to be held in Doha (Qatar), where documented T_w_ are in excess of 31°C.

Although there has been significant scientific research into the consequences of hot environmental conditions on athletes’ health and performance during terrestrial sporting activities and the development of subsequent large-scale protective guidelines (Armstrong et al., 2007), comparatively little attention has been paid to the safety and performance implications of competing in hot aquatic environments (Macaluso et al., 2011; Hue et al., 2013, 2015). Similar to cases in terrestrial sports during hot and humid conditions (Gamage et al., 2020), aquatic competition in high-risk conditions can have tragic consequences. This perspective article discusses current research and challenges in this context to stimulate the development of further research and policies to support athlete health during open-water swimming competition.

## Physiological and Performance Consequences

In comparison to terrestrial exercise, the key change in heat loss dynamics during swimming is the blunting of evaporative heat loss. In the midst of only a small contribution of evaporative heat loss (McMurray and Horvath, 1979) and the greater heat capacity of water compared to air, convection becomes a primary heat loss avenue during swimming in warm water (Nadel et al., 1974). Thus, the skin-to-water temperature gradient is a critical factor that determines the rate of body heat loss, with the gradient becoming narrower (i.e., less conducive for heat loss) at warmer T_w_ (McMurray and Horvath, 1979). A high metabolic rate (oxygen consumption [V̇O_2_] often exceeding 3.2 l/min at typical competition pace [Holmér, 1972; Toussaint et al., 1990; Zacca et al., 2020]) is also evident in elite open-water swimmers for long durations (≥60 min). Moreover, prolonged swimming in warm water is associated with elevated sweat rates (often exceeding 1 l/h; Macaluso et al., 2011; Hue et al., 2013). Elevated skin temperature during warm water swimming (Costill et al., 1967; McMurray and Horvath, 1979) is accompanied by a peripheral vasodilatory response, creating further competition for blood volume with the contracting muscles (Sawka et al., 2011). This competition for blood supply and concurrent loss of bodily fluid volume induces meaningful cardiovascular strain during exercise (Sawka et al., 2011).

More coordinated research has focused on low core temperature responses during cold water swimming exposure (T_w_ of ~10–16°C; Saycell et al., 2018). Conversely, a high core temperature response to exercise increases the risk of exertional heat illness (Armstrong et al., 2007). Some published reports suggest that rectal (T_re_) and gastrointestinal (T_gi_) temperature may not be consistently dangerous during simulated 5 km performance in T_w_ of 32.0°C (end-event T_re_ of 38.0°C; Macaluso et al., 2011) and competitive 10 km performance in T_w_ of 28.1°C (end-event T_gi_ 38.3°C; Hue et al., 2015), with either none or very little in-race cold fluid consumption. Bradford and colleagues (Bradford et al., 2013) reported that the end-event T_re_ during a simulated 120 min (similar duration to a 10 km event) swim in 32°C water was on average, 38.4°C for 22 competitive swimmers. The variation in the dataset (38.4 ± 0.8°C) highlighted that some athletes well exceeded a peak T_re_ of 39.0°C. While it is acknowledged that these core temperatures may not be problematic for some elite athletes (Racinais et al., 2019), it is still generally considered to be high risk for exertional heat illness (Roberts et al., 2021). Additionally, there are other published case reports of elite swimmers exceeding a T_gi_ of 39.0°C, and even recording close to 40.0°C during competitive swims ranging from 5 to 10 km in T_w_ up to 32°C (Bradford et al., 2019). However, methodological issues relating to appropriate depth of T_re_ sensors (Macaluso et al., 2011) as opposed to common recommendations (Lee et al., 2010), inconsistent lead-in time for the consumption of T_gi_ sensors (Hue et al., 2015) against common guidelines (Wilkinson et al., 2008), or the reporting of minimal study details (conference proceeding; Bradford et al., 2013) make it challenging to draw consistent conclusions regarding the core temperature response to warm-water competitive swimming. Future research that reports the thermal strain of athletes during competition in warm water will help move the field forward by informing and calibrating ongoing heat policy work.

While the understanding of hot and humid conditions effect upon terrestrial endurance performance is well-established (Guy et al., 2015), the effect of warm-water upon open-water swimming performance in the scientific literature is not well reported. There is some evidence that swim performance in competitive male athletes is degraded in 32°C T_w_ compared to 27°C T_w_ (~4%; Macaluso et al., 2011). Baldassarre et al. (2019) recently reported the pacing profiles of the most successful open-water swimmers in major international 10 km races showing a negative pacing profile over the course of a race, with a significant increase in speed over the last quarter of races. Mean speed over a 10 km race was 1.48 m/s and 1.38 m/s for men and women, respectively (Baldassarre et al., 2019). However, this data set did not examine the effect of T_w_. Of interest is the official results from the 2020 Olympic Games in Tokyo where official T_w_ 2 h prior to race start were 29.3°C and 29.5°C for women and men races, respectively. These data do not tend to demonstrate the same extent of negative pacing as observed in Baldassarre et al. (2019), although the prevailing T_w_ did not appear to confer substantial decrements in pace across the race. However, a much larger dataset would be required to empirically confirm the effect of T_w_ upon prolonged open-water swimming performance.

## Measures To Improve Swimmer Health During Competition in Warm Water

### Pre-race

#### Preparatory Swimming in Warm Water

Repeated exposure to environmental heat during exercise training is considered the best preparation for terrestrial competition in hot conditions (Armstrong et al., 2007; Périard et al., 2015). Extensive research is available about the beneficial effects on thermoregulatory, cardiovascular, metabolic, perceptual, and performance outcomes following running and cycling training in the heat (Périard et al., 2015). However, very little is known about the adaptations associated with repeated active swim training in warm water. Hue et al. (2007) reported that 8 days of swim training in warm water (30°C) did not significantly change 400-m time-trial performance 10 days after the final training session, but there was a significant improvement (10%) 30 days after the final session. Bradford et al. (2015) failed to observe clear physiological adaptations or performance changes in competitive swimmers following seven swim training sessions in warm water (33°C). The authors suggested that a lack of cardiovascular adaptations could relate to the lower orthostatic stress in comparison to upright exercise (running and cycling) during the short-term program (Bradford et al., 2015). Future studies likely need to manipulate training parameters to facilitate potential positive adaptations, since the adaptation process to repeated heat training is sensitive to the duration and intensity of both environmental exposure and exercise protocol (Chalmers et al., 2014). Although untested, swimmers may obtain greater heat-related adaptations from a cross-training approach (i.e., cycling or running; Bradford et al., 2015), but the benefit of this approach for swimming-specific performance is uncertain. While an expansion in plasma volume (hallmark acclimation adaptation) is likely to be beneficial for cardiovascular stability during warm-water swimming, the increase in sweat rate that typically accompanies heat acclimation protocols (Périard et al., 2015) is unlikely to be meaningful for swimming in warm water since evaporative heat loss only plays a lesser role in comparison to terrestrial sports. Therefore, the cost–benefit tradeoff of cross-training must be carefully considered. Post-exercise hot water immersion/sauna may be an attractive means for swimmers to induce some heat acclimation protective adaptations while having less disruption to regular training (Casadio et al., 2017). This suggestion, however, has yet to be empirically tested in a swim training context.

#### Pre-race Risk Stratification

Current practice for determining the risk of heat illness during competitive open-water swimming is based only on event cancellation once a maximum allowable T_w_ is recorded (Fédération Internationale de Natation (FINA), 2020). An overarching international policy is set by FINA, but local governing organizations can choose to be guided by their own policy when hosting events or when their representative athletes are competing internationally. The level of protection associated with race cancellation that current FINA international (T_w_ of 31°C) or local governing body [e.g. a T_w_ of 29.45°C (Bradford et al., 2019)] is either unclear or not reported. While T_w_ is the predominant environmental factor that determines thermal stress during swimming, it neglects other environmental factors (air temperature, humidity, wind speed, and solar radiation) that might contribute to thermal stress to some extent. Moreover, in response to observed T_w_, current policy does not implement graded risk stratification categories (i.e., low, moderate, high, or extreme) and risk-mitigation strategies prior to the race cancellation threshold, as is commonly done in many other sport and exercise settings (Armstrong et al., 2007; Sports Medicine Australia (SMA), 2021). Quantifying the level of risk would facilitate decisions by organizations and athletes to take necessary precautions. These could consist of adding cooling interventions, changing the start time to effect T_w_, or shortening the course lap length to provide more opportunity for visually checking athletes and provide greater access to cold fluids at feeding stations. It is also reasonable to expect that the level of risk associated with different T_w_ may be different during 5, 10, and 25 km events due to differences in exposure duration and metabolic heat production, but this is not currently considered by the current international policy (Fédération Internationale de Natation (FINA), 2020).

Stratified risk for competition cohorts can be determined through biophysical modeling that estimates human heat transfer in response to both environmental and typical individual (i.e., exercise intensity) factors based upon readily available environmental and race-specific details. These biophysical modeling tools require foundational data relating to heat transfer avenues (convection, evaporation, radiation, conduction) and typical individual factors (metabolic rate, clothing, body size). In some instances, these factors are less well-established or understood in comparison to common terrestrial activities. Briefly, a primary challenge during biophysical modeling for open-water swimming is determining the most appropriate convective heat transfer coefficient (Nadel et al., 1974; Boutelier et al., 1977; Yermakova and Montgomery, 2018). The comparative effect of different T_w_ upon self-paced swimming speed by trained athletes during competition (simulated or actual) over distances relevant for international competition (≥5 km) also remains underreported (Macaluso et al., 2011). The absence of these data influences the modeling of metabolic rate (i.e., heat production) during biophysical modeling, alongside the known relationships between speed, V̇O_2_, and efficiency (Holmér, 1972; Toussaint et al., 1990; Zacca et al., 2020). Conceivably, T_w_ could be publicly reported by FINA alongside official race results. Moreover, the effect of environmental solar radiation on the prevailing heat load of an athlete during open-water swimming remains uncertain, influencing the biophysical modeling of radiative heat transfer. A pilot laboratory study reported that an additional radiant heat load (~400–800 W/m^2^) did not have a meaningful impact upon T_re_ in comparison to the absence of additional radiant load, although this was based on between-study comparisons (Bradford et al., 2013). Between-study comparisons are limited by confounding variables related to, but not limited to, differences in body size, metabolic heat production during self-paced performance, and participant sex. Future research and reporting in these areas will help advance the field of biophysical modeling in open-water swimming.

The effective implementation of any policy that is determining risk before or during an event is constrained by the reliability and validity of measured parameters. Current international practice is to standardize the measurement of T_w_ at a depth of 40 cm (Fédération Internationale de Natation (FINA), 2020), but there is no standardization of measurement instrument, which can have important implications for the determination and categorization of risk in a sport and exercise setting (Cooper et al., 2017). This is important since the management of heat illness in open-water swimming competition occurs arguably between only a ~ 4°C zone of T_w_ (29°C–33°C), indicating that T_w_ changes of even 0.1°C may conceivably have meaningful implications.

### In-race Cooling

In-race hydration is a common and feasible intervention during open-water swimming. Course feeding stations offer key strategic rehydration opportunities but require swimmers to make decisions between the necessary time required to consume cold beverages and the additional performance improvement or cooling effect that it may confer. While not largely reported, there appears to be a reluctance by some elite open-water swimmers to undertake substantial fluid replacement during competition (T_w_ of ~28.1°C; Hue et al., 2015). This is possibly due to athletes not valuing the additional time required to consume cool fluids (Hue et al., 2015) or gastrointestinal comfort concerns, but little attention has been paid to this question.

A review by Jay and Morris (2018) highlighted that the independent effect of cold water or ice slurry ingestion upon core temperature during terrestrial exercise is dependent on the prevailing environmental conditions. Cold fluid stimulates thermoreceptors in the stomach that may ultimately reduce the sweat rate response to exercise (Jay and Morris, 2018). Therefore, cold beverages may not always result in a net cooling benefit during exercise due to the potential for a concurrent reduction in sweat rate that reduces evaporative heat loss to at least the equivalent amount of the additional internal heat loss with the ingested cold fluid (Jay and Morris, 2018). However, in the context of swimming, evaporative heat loss plays a lesser role in comparison to terrestrial sporting events (McMurray and Horvath, 1979), and therefore, it is likely that the internal cooling benefit of drinking cold water during a swim race will outweigh the potential for any reductions in evaporative potential. Moreover, since much of the skin is continuously wet during swimming, any reductions in physiologically driven skin wettedness (*via* a reduction in sweating) that might occur with cold fluid ingestion may not have any meaningful effect upon evaporative potential during a race. An important consideration for warm water races could be to manipulate the length of each course lap, which will ultimately provide more regular access to feeding pontoons. This might encourage swimmers to consume small regular boluses of cold fluid, reducing the risk of gastric discomfort associated with potential reductions in gastric emptying rates (Anvari et al., 1995).

Like all cooling strategies, scientific evidence in the swimming literature is sparse. Hue et al. (2013) observed nine internationally-ranked swimmers during four 5 km training swims at a fixed pace while consuming either 190 ml of neutral (27°C) or cold fluid (1°C) each 1 km. The cold-fluid intervention mitigated the rise in T_gi_ (≥ ~ 0.4°C) in comparison to the neutral-water trial during evening training, but not morning training. The authors theorized that this could be due to the athletes experiencing slightly greater thermal strain (starting T_gi_) and thermal stress (higher T_w_ of 29.1°C vs. 29.9°C) during the evening (Hue et al., 2013). Importantly, the athletes were only provided the ingestible temperature sensor 3 h prior to the training session (Hue et al., 2013), which is much shorter than commonly recommended [~8 h; (Wilkinson et al., 2008)] to avoid measurement issues with cold fluid consumption.

Hue et al. (2015) also examined the effect of fluid consumption in eight internationally-ranked open-water swimmers during two 5 km trials at a fixed race pace in T_w_ of ~28.6°C. The trials involved the swimmers ingesting either 190 ml of neutral (28°C) or cold (1°C) fluid at each 1 km interval (5 drinks in total). The rate of T_gi_ change was higher during the 5 km trial in the neutral-fluid consumption group in comparison to the cold-fluid consumption group (difference of ~1°C/h). The reason for such a large between-group discrepancy is unclear. Moreover, the same swimmers were observed during a sanctioned 10 km event in warm-water (T_w_ of 28.1°C) where they could consume neutral temperature (28°C) water *ad-libitum* at every 2 km interval. Notably, during the sanctioned competition event, athletes freely consumed only ~10% of the fluid that was consumed during the intervention trials (113 ml vs. 950 ml). This is an important consideration for the implementation of heat-related risk mitigation strategies, since when given a choice, open-water swimmers may not choose or be well-practiced at drinking larger volumes of cold fluid during a race that can result in meaningful changes to core temperature. This might relate to race tactics and the potential time-loss associated with the rehydration process.

Using known partitional calorimetry principles for determining change in body heat storage (Cramer and Jay, 2019), we predict that a heat-stressed (core temperature of 39.0°C) elite swimmer during a 10 km event will need to consume ~400 *g* of 5°C fluid to elicit a meaningful reduction in core temperature (≥0.2°C) in comparison to not drinking. This assumes that the cold fluid consumption will have a negligible impact upon evaporative heat loss potential. This volume of fluid seems very achievable when consumed in small boluses, especially if a course is manipulated to allow more regular access to feeding pontoons. Further systematic research is needed that marries an ecologically valid volume of fluid consumption during simulated controlled conditions. Due to the relative contextual difficulty and consequences of rehydrating during competitive swimming in comparison to running and cycling, athletes may disregard in-race hydration cooling recommendations that are not mandatory if individual performance is perceived to be substantially impacted. In this instance, a compulsory hydration stop could address this issue.

### Post-race Cooling

While the literature is sparse in a swimming-specific context for post-race cooling, insights can be gleaned from comprehensive reviews (McDermott et al., 2009) and position statements (Armstrong et al., 2007; Casa et al., 2015) from other sports. The post-race context is generally more generic in comparison to the in-event context across many sports. Cold-water immersion is considered the best strategy for quickly cooling a heat-stressed athlete (Casa et al., 2015).

## Conclusion

This perspective article highlights the challenges and opportunities to develop evidence-based policies that mitigate risk during warm-water swimming competition. Future work is needed to advance knowledge on thermal strain and performance during warm-water competition, as well as evaluation of preparatory and in-race cooling strategies. Open-water swimming warm-water race policies would benefit from risk stratification (low, moderate, high, extreme) for a given race distance according to a holistic representation of prevailing environmental conditions, rather than just a single T_w_ threshold for the cancellation of all events.

## Data Availability Statement

The original contributions presented in the study are included in the article/supplementary material, further inquiries can be directed to the corresponding author.

## Author Contributions

SC, GS, IM, and OJ contributed to the conception and writing of the manuscript. All authors contributed to the article and approved the submitted version.

## Conflict of Interest

SC and OJ have a financial consultancy agreement with Swimming Australia but received no honoraria for this paper. Hence, the authors declare that this article was conducted in the absence of any commercial or financial relationships that could be construed as a potential conflict of interest.

## Publisher’s Note

All claims expressed in this article are solely those of the authors and do not necessarily represent those of their affiliated organizations, or those of the publisher, the editors and the reviewers. Any product that may be evaluated in this article, or claim that may be made by its manufacturer, is not guaranteed or endorsed by the publisher.
